# Reducing Late Dysphagia for Head and Neck Cancer Survivors with Oral Gel: A Feasibility Study

**DOI:** 10.1007/s00455-019-10018-9

**Published:** 2019-05-09

**Authors:** Julie Killerup Kaae, Marie Louise Spejlborg, Ulrik Spork, Kristine Bjørndal, Jesper Grau Eriksen

**Affiliations:** 1 Department of Clinical Research, University of Southern Denmark, J.B. Winslows Vej 19.3, 5000 Odense C, Denmark; 2 Department of Oncology, Odense University Hospital, Kløvervænget 19, Entrance 85, 5000 Odense C, Denmark; 3 Salient Pharma IvS, Taarbaeck Strandvej 108A, 2930 Klampenborg, Denmark; 4 Department of Otolaryngology – Head and Neck Surgery, Odense University Hospital, J.B. Winslows Vej 4, 5000 Odense C, Denmark; 5 Department of Experimental Clinical Oncology, Aarhus University Hospital, Palle Juul-Jensens Boulevard 99, 8200 Aarhus, Denmark

**Keywords:** Dysphagia, Head and neck cancer, Radiotherapy, Saliva substitute, Deglutition, Deglutition disorders

## Abstract

Dysphagia is a significant late morbidity following treatment with radiotherapy (RT) for head and neck squamous cell carcinomas (HNSCC). The purpose of this feasibility study was to test a gel-based saliva substitute to reduce the subjective assessment of dysphagia while eating food items varying in size and texture. Eligible study subjects treated with curative intended RT and suffering from dysphagia and xerostomia were recruited from the outpatient clinic during a two-month period. Nineteen subjects consented to participation and completed the EORTC QLQ-H&N35 questionnaire and three test meals. A Numeric Rating Scale (NRS) was used for subjective assessment of dysphagia during all test meals. All data on patient, tumor, and treatment characteristics were obtained from the Danish Head and Neck Cancer (DAHANCA) database. NRS data suggested reduction of dysphagia after application of the oral gel. The swallowing dysfunctions, discomfort while swallowing (*p* = 0.008), stuck food items (*p* = 0.02), and multiple attempts of clearing the throat (*p* = 0.05), improved significantly for soft and regular items. Both small- and large-sized food items were tested. EORTC QLQ-H&N35 showed improvement regarding eating problems (*p* = 0.03) and social eating (*p* = 0.02). No episodes of food aspiration were recorded during the test meals. Late dysphagia reduces QOL and is an important morbidity following RT. In this feasibility study, the oral gel was able to reduce dysphagia while eating soft and selected regular food items. Eating-related EORTC QLQ-H&N35 items also improved, indicating a beneficial reduction in dysphagia after application of the oral gel.

**Electronic supplementary material:**

The online version of this article (10.1007/s00455-019-10018-9) contains supplementary material, which is available to authorized users.

## Introduction

Survival after head and neck squamous cell carcinomas (HNSCC) has greatly improved within the last decades partly due to improved treatment options. Radiotherapy (RT) remains the main treatment modality for HNSCC, to obtain tumor control and prevent loco-regional failure. Treatment morbidity, acute as well as late, is, however, a recurrent problem for all HNSCC patients.

Dysphagia, or swallowing dysfunction, is predominant during and shortly after RT [1]. Acute dysphagia is an acute inflammatory response characterized by tissue erythema, mucositis, edema, xerostomia, and pain, which often gradually improves within the first 6 months after treatment [1, 2]. Late dysphagia is defined as persistent swallowing problems beyond 6 months post-treatment, and is characterized by damage to the soft tissue and fibrosis, which may lead to alteration of the tube-like compartment essential for the swallowing function [3].

Curative intended RT for HNSCC involves irradiation of the salivary glands and oral cavity, and both acute and late normal tissue morbidity are inevitable [4]. Approximately 50% of all HNSCC survivors develop some degree of persistent or late dysphagia [5, 6]. A correlation has been found between frequency and intensity of radiation-induced dysphagia and the dose volume relationship for organs at risk, such as the supraglottic region and larynx [7, 8].

Management of late dysphagia remains an important clinical challenge, and radiation-induced dysphagia is associated with compromised health-related quality of life (QOL) [1, 2, 9–12]. The functional impairment has a negative impact on social eating, particularly within the first year after treatment for HNSCC [13]. Social eating is an important aspect of late dysphagia, with up to one quarter of the HNSCC survivors feeling displeasure when eating in social settings up to 12 months after treatment [14]. The inability to swallow normally can be socially restraining, both physically and mentally, and adjustments towards new eating routines must be established before feeling at ease in social situations. Considerations about dietary habits, e.g., food content, texture, and careful eating skills, are essential to obtain sufficient nutrition and avoid complications such as aspiration [15].

The purpose of this feasibility study was to test the efficacy of a newly devolved gel-based saliva substitute with regard to (1) subjective reduction of dysphagia, (2) increased variety in standardized food items, and (3) improvement of quality of life for HNSCC survivors.

## Methods

### Study Subjects

Eligible candidates were HNSCC patients treated with primary curative intended RT (66–68 Gy) without loco-regional recurrence. Self-reported radiation-induced xerostomia and dysphagia had to be present, and scored according to the Danish Head and Neck Cancer (DAHANCA) group’s toxicity scale. This scale is comparable with the Late Effects of Normal Tissues (LENT)-Subjective, Objective, Management, and Analytic (SOMA) scale [16]. The LENT-SOMA scale was developed by the Radiation Therapy Oncology Group (RTOG) and the European Organization for Research and Treatment of Cancer (EORTC) group for assessment and grading of late effects after RT, and has been incorporated into the Common Terminology Criteria for Adverse Events (CTCAE). The DAHANCA group has developed an observer-based toxicity tool on the basis of this scale. Overall treatment was completed at least 6 months prior to participation to avoid influence of acute toxicity. Eligible study subjects were identified in the outpatient clinic by the study investigator, JKK.

### Gel-Based Saliva Substitute

The oral gel used in the study was developed to relieve symptoms associated with dysphagia including xerostomia. The gel was water based, edible, and free from flavored additives. Constituents were primarily hydroxypropyl methylcellulose K200 M (HPMC) and polyethylene oxide wsr-301 (PEO), which are edible polymers providing gel texture, saliva like ‘stringiness,’ and mucosal adherence without the need for taste masking. The properties of the gel were chosen with the purpose of lubricating and adding moisture to the oral cavity, thereby reducing friction and easing the swallowing process and reducing side effects of xerostomia. Both potassium sorbate and malic acid were added to create a buffer system to avoid microbiological demotion as well as maintaining a pH value of 6.0. The gel was distributed in 50-ml dispensers and gave approximately 3 ml per deposit. Gel was deposited on a teaspoon and swallowed prior to eating.

### The Test Meal

The test meal consisted of six standardized food items ranging in texture from pureed, minced, and soft to regular food. All items were selected by the hospital’s head and neck cancer dedicated dietician, and the test meal was served in small and large bites, respectively, with increasing level of difficulty (for illustration of the test meal see Supplementary material). Each food item was evaluated on a Numeric Rating Scale (NRS) ranging from zero to ten, with a low score indicating severe difficulties while eating and a high score indicating no difficulties. All test meals were prepared and delivered by the hospital catering center.

### Study Setting

Prior to commencing the study, clearance was sought from the Regional Committees on Health Research Ethics for Southern Denmark (S-20160101), the Regional Danish Data Protection Agency (16/23688), and the Danish Medicines Agency (LMST 2016062991). All study subjects received oral and written information before informed consent was obtained.

The oral gel was tested in a joined setting with four to eight subjects per group. The test period was 1 week. The first and second test meals were tested on the first test day, and the third test meal on the second test day, 1 week later, to test for intra-judge reliability. The first test meal was evaluated without the oral gel, whereas the second meal was evaluated while using the gel. Subjects were instructed to use the gel (3 ml approximately) before eating the very first food item and re-apply when needed throughout the test meal on an individual basis. Dysphagia and QOL was evaluated using the European Organization for Research and Treatment of Cancer (EORTC) questionnaire module for head and neck cancer (QLQ-H&N35) [17, 18]. No intake of water was allowed immediately prior to or during the test meals. This approach was chosen to better evaluate the effectiveness of the oral gel. All subjects were provided with oral gel for use at home in-between the test days, and encouraged to test it on everyday food items in their habitual diet. On the second test day, the third test meal was evaluated with the gel and the EORTC QLQ-H&N35 questionnaire was completed for the last time. A head and neck surgeon was on call during all test days in case of food aspiration.

### Sialometry

Unstimulated whole salivary flow was measured prior to initial testing, to determine salivary flow rate and the degree of xerostomia. Subjects were seated in an undisturbed and calm environment, and sialometry was performed by passively spitting into a pre-weighted plastic tube for 5 min [19]. Afterwards, the tube was weighted again, and the flow rate determined as ml/five min, taking into account that saliva density is approximately one mg/ml. The electronic weight, Metler Toledo PG4002-S, was used for measurements.

### Data Analyses

Patient and tumor characteristics were described using Pearson Chi-square and Spearman correlations. NRS scores for each subjective assessment of the three test meals were recorded, and the scores were analyzed using the ANOVA test. For the EORTC QLQ-H&N35 module, only the scales most relevant for the purpose of this study were analyzed, i.e., swallowing, dry mouth, sticky saliva, and social eating. The comparison of scores was tested with Wilcoxon Signed Rank test. Data management and analysis were performed using SPSS (IBM SPSS, version 24, Chicago, IL, USA).

## Results

### Study Subjects

Thirty-six consecutive study subjects were asked to participate from April to May in 2017. Twelve declined participation, and five withdrew consent after the first test meal. Nineteen subjects completed the study (Table 1). The subjects participating only differed significantly from those declining to participate with regard to performance status. A lower performance status was observed at the time of diagnosis for the study subjects. All study subjects had received primary curative intended RT (66–68 Gy) for HNSCC located in the oral cavity (5%), pharynx (84%), or larynx (11%). Locally advanced cancer was most prevalent, and subjects had completed treatment within 6–50 months prior to the study.

**Table 1:** Characteristics of eligible study participants

	Participating (*n* = 19)		Not participating (*n* = 17)		*p* value
Sex, *n* (%)					
Male	9	(47)	12	(71)	0.2
Female	10	(53)	5	(29)	
Age, years					
Median [range]	60	[46–80]	66	[49–80]	0.7
Tumor location, *n* (%)					
Larynx	2	(11)	1	(6)	0.5
Pharynx	16	(84)	13	(76)	
Oral cavity	1	(5)	3	(18)	
Primary RT, *n* (%)					
Curative intended	19	(100)	16	(94)	0.5
Performance status, *n* (%)					
0–1	19	(100)	12	(71)	0.04
≥ 2	0		5	(29)	
Comorbidity, *n* (%)					
No comorbidity	4	(21)	6	(35)	0.5
≥ 1 comorbidity	15	(79)	11	(65)	
Tumor stage, *n* (%)					
Early	7	(37)	2	(12)	0.1
Locally advanced	12	(63)	15	(88)	
Months after RT					
Median [range]	19	[6–50]	23	[0–39]	0.5
RT dose, Gy					
Median [range]	66	[66–68]	66	[66–76]	0.5

Unstimulated sialometry found a mean whole salivary flow rate of 0.1 ml/min. This is below the defined threshold for unstimulated whole saliva flow for healthy individuals with a normal flow rate of 0.3–0.5 ml/min [20]. All participants had subjective complaints of xerostomia, and for ten subjects the unstimulated whole saliva flow rate was very low (< 0.1 ml/min).

### Evaluation of the Gel-Based Saliva Substitute

**Fig. 1:**
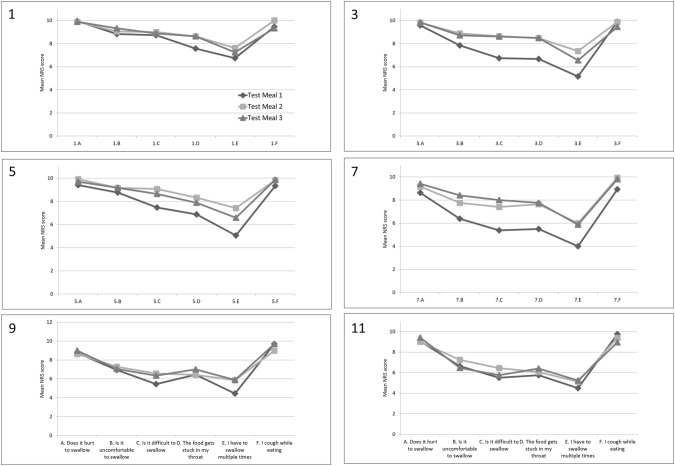
Subjective assessment of small food items. *NRS* numeric rating scale. Small food items, 1; small pureed item (yoghurt), 3; small minced item (gratin), 5; small soft item (white bread), 7; small regular item (rye bread), 9; small regular item (crisp bread), 11; small regular item (meat). Subjective assessment: A. Does it hurt to swallow; B. Is it uncomfortable to swallow; C. Is it difficult to swallow; D. The food gets stuck in my throat; E. I have to swallow multiple times; F. I cough while eating

**Fig. 2:**
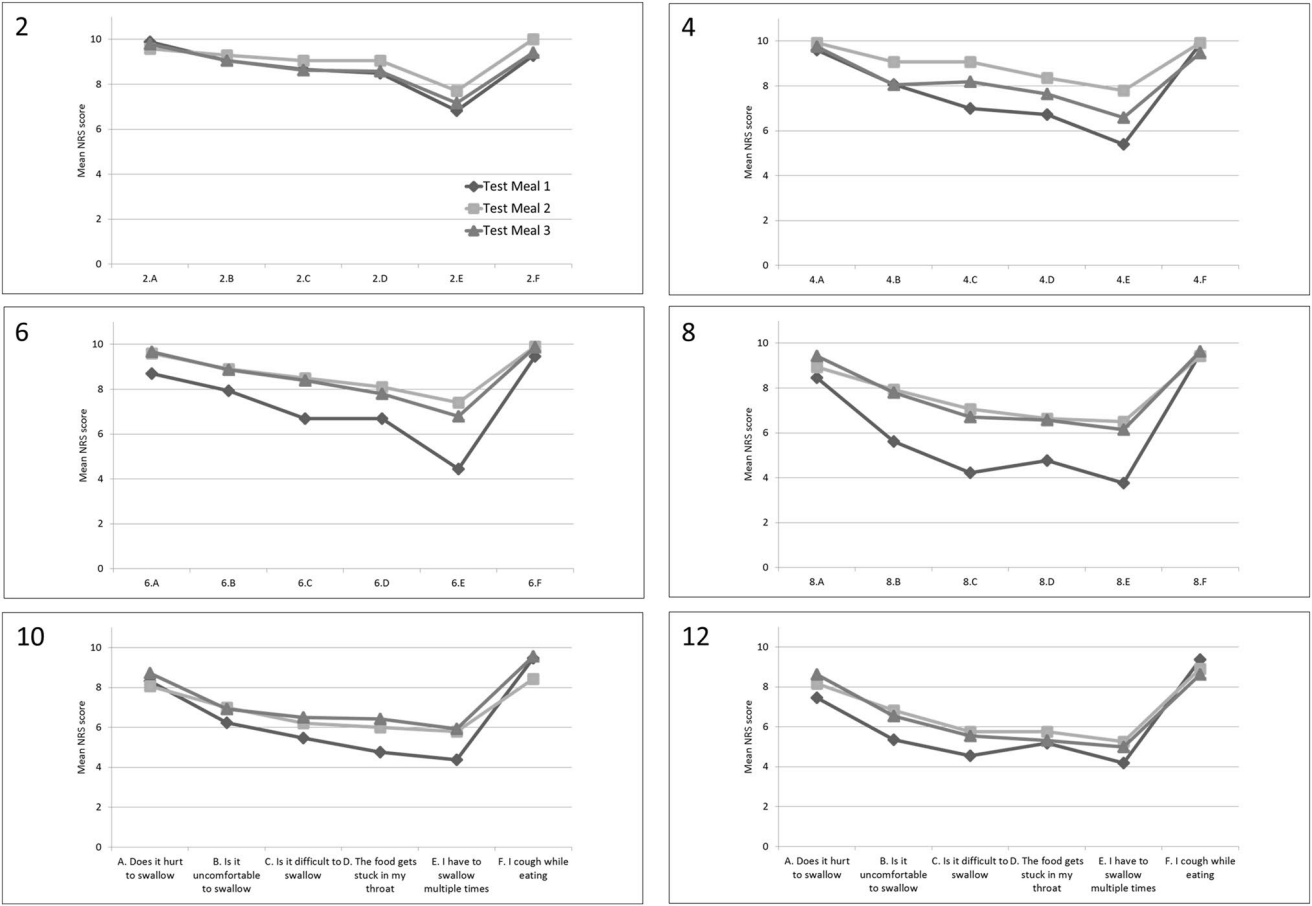
Subjective assessment of small food items. *NRS* numeric rating scale. Large food items, 2; large pureed item (yoghurt), 4; large minced item (gratin), 6; large soft item (white bread), 8; large regular item (rye bread), 10; large regular item (crisp bread), 12; large regular item (meat). Subjective assessment: *A*. Does it hurt to swallow; *B*. Is it uncomfortable to swallow; *C*. Is it difficult to swallow; *D*. The food gets stuck in my throat; *E*. I have to swallow multiple times; *F*. I cough while eating

After application of the oral gel, improvement was observed for the subjective assessment of swallowing. Subjects reported a tendency towards less difficulty with the swallowing process, less obstruction of the food, no pain while eating, and less discomfort with social eating (Figs. 1 and 2).

A significant improvement in mean NRS score was seen for the assessment of some of the regular food items. For the small regular food items (i.e., rye bread), the swallowing process increased with 2.62 scores after application of the gel at the third test meal compared to the first (*p* = 0.008) indicating an improvement of the swallowing function (Table 2). A similar improvement of 2.48 scores, from 4.23 at the first test meal to 6.71 at the third test meal (*p* = 0.004), was seen for the large items (Table 3). All six standardized food items showed a positive trend for food not being stuck in the throat when applying the gel, regardless of the food size.

**Table 2:** Mean NRS score for small food items

Small food items	Test meal 1		Test meal 2		Test meal 3		*p* value
	*M*	(95% CI)	*M*	(95% CI)	*M*	(95% CI)	
Pureed (yoghurt)							
Does it hurt to swallow?	9.95	(9.84–10.06)	9.89	(9.74–10.05)	9.89	(9.67–10.12)	0.87
Is it uncomfortable to swallow?	8.84	(7.47–10.22)	9.05	(7.86–10.25)	9.32	(8.20–10.43)	0.85
Is it difficult to swallow?	8.74	(7.53–9.95)	9.00	(7.84–10.16)	8.89	(7.74–10.05)	0.95
Does the food get stuck in your throat?	7.58	(5.98–9.18)	8.63	(7.46–9.80)	8.63	(7.42–9.85)	0.41
Do you have to swallow multiple times?	6.74	(5.08–8.40)	7.63	(6.13–9.13)	7.26	(5.77–8.75)	0.69
Do you cough while eating?	9.47	(8.37–10.58)	10.0		9.32	(8.31–10.32)	0.47
Minced (jelly bread)							
Does it hurt to swallow?	9.72	(9.35–10.10)	9.82	(9.55–10.10)	9.83	(9.48–10.18)	0.52
Is it uncomfortable to swallow?	7.89	(6.26–9.52)	8.88	(7.99–9.77)	8.72	(7.63–9.81)	0.39
Is it difficult to swallow?	6.72	(4.98–8.46)	8.65	(7.53–9.77)	8.61	(7.69–9.53)	0.05*
Does the food get stuck in your throat?	6.67	(4.93–8.41)	8.47	(7.38–9.56)	8.50	(7.68–9.32)	0.05*
Do you have to swallow multiple times?	5.22	(3.64–6.81)	7.35	(6.19–8.52)	6.56	(5.36–7.75)	0.05*
Do you cough while eating?	9.89	(9.65–10.12)	9.88	(9.71–10.05)	9.44	(8.76–10.13)	0.23
Soft (white bread)							
Does it hurt to swallow?	9.41	(8.73–10.09)	9.92	(9.73–10.10)	9.71	(9.20–10.21)	0.37
Is it uncomfortable to swallow?	8.76	(7.98–9.55)	9.17	(8.57–9.76)	9.18	(8.47–9.89)	0.48
Is it difficult to swallow?	7.47	(6.12–8.82)	9.08	(8.39–9.77)	8.65	(7.74–9.55)	0.07
Does the food get stuck in your throat?	6.88	(5.22–8.55)	8.33	(7.14–9.52)	7.88	(6.89–8.88)	0.31
Do you have to swallow multiple times?	5.06	(3.31–6.81)	7.42	(5.67–9.16)	6.59	(5.37–7.81)	0.21
Do you cough while eating?	9.35	(8.11–10.60)	9.83	(9.59–10.08)	9.88	(9.71–10.05)	0.46
Regular (rye bread)							
Does it hurt to swallow?	8.63	(7.38–9.87)	9.18	(8.30–10.05)	9.38	(8.65–10.10)	0.45
Is it uncomfortable to swallow?	6.38	(4.70–8.05)	7.76	(6.34–9.19)	8.31	(7.39–9.24)	0.08
Is it difficult to swallow?	5.38	(3.63–7.12)	7.41	(6.43–8.39)	7.94	(6.94–8.94)	0.008*
Does the food get stuck in your throat?	5.50	(3.70–7.30)	7.65	(6.54–8.75)	7.75	(6.79–8.71)	0.02*
Do you have to swallow multiple times?	4.00	(2.44–5.56)	6.00	(4.69–7.31)	5.81	(4.69–6.93)	0.05*
Do you cough while eating?	8.94	(7.49–10.39)	9.94	(9.82–10.07)	9.81	(9.52–10.10)	0.16
Regular (crisp bread)							
Does it hurt to swallow?	8.75	(7.38–10.12)	8.64	(6.98–10.30)	9.00	(7.63–10.37)	0.93
Is it uncomfortable to swallow?	6.94	(5.25–8.63)	7.29	(5.48–9.09)	7.06	(5.38–8.74)	0.96
Is it difficult to swallow?	5.44	(3.82–7.05)	6.57	(4.75–8.39)	6.35	(4.78–7.93)	0.56
Does the food get stuck in your throat?	6.44	(4.89–7.98)	6.43	(4.68–8.18)	7.00	(5.62–8.38)	0.81
Do you have to swallow multiple times?	4.44	(3.06–5.81)	5.86	(4.37–7.34)	5.88	(4.37–7.39)	0.24
Do you cough while eating?	9.69	(9.15–10.23)	9.00	(7.60–10.40)	9.71	(9.27–10.14)	0.37
Regular (meat)							
Does it hurt to swallow?	9.06	(8.14–9.98)	9.00	(7.78–10.22)	9.41	(8.62–10.21)	0.79
Is it uncomfortable to swallow?	6.63	(5.20–8.05)	7.25	(5.56–8.94)	6.47	(4.64–8.30)	0.76
Is it difficult to swallow?	5.50	(3.88–7.12)	6.44	(4.91–7.97)	5.76	(4.16–7.37)	0.66
Does the food get stuck in your throat?	5.75	(3.95–7.55)	6.06	(4.46–7.66)	6.41	(4.91–7.91)	0.83
Do you have to swallow multiple times?	4.50	(2.85–6.15)	5.13	(3.52–6.73)	5.24	(3.73–6.74)	0.76
Do you cough while eating?	9.75	(9.44–10.06)	9.38	(8.18–10.57)	8.94	(7.59–10.30)	0.53

*M* mean

*Statistically significant *p* value

**Table 3:** Mean NRS score for large food items

Large food items	Test meal 1		Test meal 2		Test meal 3		*pvalue*
	*M*	(95% CI)	*M*	(95% CI)	*M*	(95% CI)	
Pureed (yoghurt)							
Does it hurt to swallow?	9.89	(9.73–10.05)	9.59	(8.96–10.22)	9.79	(9.35–10.23)	0.61
Is it uncomfortable to swallow?	9.06	(8.08–10.03)	9.29	(8.45–10.14)	9.05	(7.89–10.22)	0.92
Is it difficult to swallow?	8.67	(7.62–9.72)	9.06	(8.32–9.80)	8.63	(7.44–9.83)	0.79
Does the food get stuck in your throat?	8.50	(7.49–9.51)	9.06	(8.30–9.82)	8.58	(7.38–9.77)	0.69
Do you have to swallow multiple times?	6.83	(5.30–8.37)	7.71	(6.27–9.15)	7.16	(5.73–8.59)	0.68
Do you cough while eating?	9.28	(8.10–10.46)	10.0		9.42	(8.43–10.42)	0.48
Minced (jelly bread)							
Does it hurt to swallow?	9.60	(9.14–10.06)	9.93	(9.77–10.08)	9.76	(9.38–10.15)	0.43
Is it uncomfortable to swallow?	8.07	(6.54–9.60)	9.07	(8.24–9.90)	8.06	(6.81–9.31)	0.40
Is it difficult to swallow?	7.00	(5.27–8.73)	9.07	(8.21–9.93)	8.18	(6.98–9.37)	0.08
Does the food get stuck in your throat?	6.73	(5.07–8.40)	8.36	(7.19–9.53)	7.65	(6.39–8.91)	0.23
Do you have to swallow multiple times?	5.40	(3.79–7.01)	7.79	(6.74–8.83)	6.59	(5.45–7.72)	0.03*
Do you cough while eating?	9.87	(9.67–10.06)	9.93	(9.77–10.08)	9.47	(8.84–10.10)	0.22
Soft (white bread)							
Does it hurt to swallow?	9.00	(8.02–9.98)	9.60	(8.91–10.29)	9.67	(9.09–10.25)	0.14
Is it uncomfortable to swallow?	8.20	(7.11–9.29)	8.90	(7.98–9.82)	8.87	(8.01–9.73)	0.24
Is it difficult to swallow?	6.87	(5.26–8.47)	8.50	(7.27–9.73)	8.40	(7.38–9.42)	0.06
Does the food get stuck in your throat?	6.67	(5.00–8.34)	8.10	(6.69–9.51)	7.80	(6.58–9.02)	0.24
Do you have to swallow multiple times?	4.47	(2.90–6.03)	7.40	(5.61–9.19)	6.80	(5.73–7.87)	0.004*
Do you cough while eating?	9.47	(8.66–10.27)	9.90	(9.67–10.13)	9.87	(9.67–10.06)	0.36
Regular (rye bread)							
Does it hurt to swallow?	8.46	(7.01–9.91)	8.93	(7.81–10.05)	9.43	(8.80–10.06)	0.42
Is it uncomfortable to swallow?	5.62	(3.97–7.26)	7.93	(6.74–9.12)	7.79	(6.50–9.07)	0.03*
Is it difficult to swallow?	4.23	(2.75–5.71)	7.07	(5.88–8.26)	6.71	(5.42–8.00)	0.004*
Does the food get stuck in your throat?	4.77	(2.94–6.60)	6.64	(5.33–7.96)	6.57	(5.45–7.70)	0.09
Do you have to swallow multiple times?	3.77	(2.33–5.21)	6.50	(5.22–7.78)	6.14	(5.08–7.20)	0.004*
Do you cough while eating?	9.54	(9.07–10.01)	9.43	(8.62–10.24)	9.64	(9.21–10.07)	0.89
Regular (crisp bread)							
Does it hurt to swallow?	8.31	(6.28–10.33)	8.07	(6.30–9.85)	8.71	(7.07–10.35)	0.86
Is it uncomfortable to swallow?	6.23	(4.22–8.24)	7.00	(5.17–8.83)	6.93	(5.35–8.51)	0.77
Is it difficult to swallow?	5.46	(3.67–7.25)	6.21	(4.59–7.83)	6.50	(4.94–8.06)	0.62
Does the food get stuck in your throat?	4.77	(2.75–6.79)	6.00	(4.40–7.60)	6.43	(4.85–8.01)	0.33
Do you have to swallow multiple times?	4.38	(2.70–6.07)	5.79	(4.30–7.27)	5.93	(4.62–7.24)	0.24
Do you cough while eating?	9.46	(8.78–10.14)	8.43	(6.55–10.31)	9.57	(9.03–10.11)	0.29
Regular (meat)							
Does it hurt to swallow?	7.45	(5.16–9.75)	8.17	(6.05–10.28)	8.62	(6.95–10.28)	0.67
Is it uncomfortable to swallow?	5.36	(3.23–7.49)	6.83	(4.64–9.03)	6.54	(4.51–8.57)	0.55
Is it difficult to swallow?	4.55	(2.46–6.63)	5.75	(3.78–7.72)	5.54	(3.84–7.24)	0.59
Does the food get stuck in your throat?	5.18	(2.78–7.58)	5.75	(3.70–7.80)	5.31	(3.47–7.14)	0.91
Do you have to swallow multiple times?	4.18	(2.17–6.19)	5.25	(3.33–7.17)	5.00	(3.36–6.64)	0.66
Do you cough while eating?	9.36	(8.35–10.37)	8.92	(7.28–10.55)	8.62	(7.34–9.89)	0.69

*M* mean

*Statistically significant *p* value

The same trend was seen for the assessment of clearing the throat using multiple swallowing attempts which eased with the gel (Table 3). Reduction in mean scores for multiple swallowing attempts was statistically significant for the pureed, minced, and soft food items. No significant difference was seen for the regular food items regarding multiple swallowing attempts, regardless of the food size (Tables 2 and 3). During the study period, no patients reported incidents of increased coughing or trouble breathing as possible signs of aspiration. However, as participants did not undergo fiberoptic endoscopic evaluation of swallowing (FEES), it was not possible to rule out the risk of silent aspiration.

The EORTC QLQ-H&N35 questionnaire showed improvement in QOL during the test period for the eating-related items after application of the gel. The evaluation showed less problems when swallowing regular food (*p* = 0.02), reduced episodes of choking when swallowing (*p* = 0.05), and less difficulties eating (*p* = 0.03). Social eating also improved and found that subjects had less trouble eating in front of others (*p* = 0.02) and less trouble enjoying their meals (*p* = 0.05). No significant improvement in QOL was seen for the single-items dry mouth or sticky saliva.

### Evaluation of the Test Meals

**Fig. 3:**
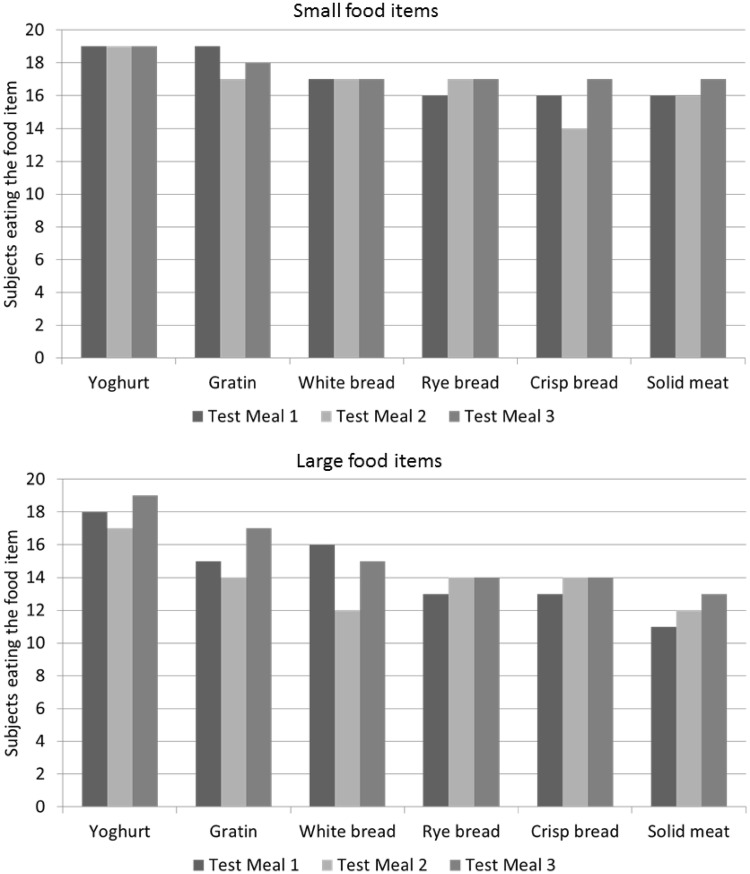
Frequency of completed food items during the test meals

The test meals were designed to challenge swallowing problems, and increased in difficulty with each mouthful. The six food items were ranked according to texture, size, and swallowing difficulties based on recommendations from the hospital’s head and neck cancer dedicated dietician [21]. When testing the smaller version of the food items during the first test meal, three subjects refrained from evaluation of the small regular (solid meat) item (Fig. 3, small food items). Six out of nineteen study subjects declined to eat and evaluate the regular large food items (rye bread and crisp bread) at the first test meal without the gel (Fig. 3, large food items). Eight subjects did not evaluate the final large regular item (solid meat) during the first test meal. The second and third test meals were both completed with the oral gel. Subjective assessment of the large food items showed a positive trend for improved swallowing function after application of the gel (*p* = 0.004); however, this was not reflected in the frequency of patients eating the difficult and large regular food (Fig. 3).

## Discussion

The purpose of this feasibility study was to examine the effects of an oral gel on perceived swallowing difficulties for HNSCC survivors while eating standardized food items. It was hypothesized that self-reported assessment of dysphagia as defined by EORTC QLQ-H&N35 would decrease after application of the oral gel. It was also hypothesized that regular food items would be less difficult to eat after application of the gel. The findings of this study partially support the hypotheses. The swallowing difficulties reported by the participants were significantly improved while using the gel for the large soft food items and selected regular food items.

RT can lead to severe functional impairments in the oral cavity and eating-related structures in the pharynx, and may result in radiation-induced dysphagia [3]. The swallowing process involves all food items to be chewed and processed with saliva to create the food bolus and avoid adhering to the palate. However, the process of managing the food bolus can be extremely difficult if reconstructive surgery or RT in the oral cavity and pharynx has been part of the cancer treatment. Both modalities can lead to restricted tongue movements, resection of teeth, reduced opening of the mouth (trismus), and bothersome scar tissue or fibrosis in the pharynx [8]. Therefore, a varied diet may be difficult to accomplish with prominent dysphagia when the diet is restricted to certain food textures [22].

Swallowing difficulties may lead to unintentional weight loss, dehydration, nutritional insufficiency, and dietary changes [11, 23]. A new routine has to be established, for both the HNSCC survivors and their families, which has a great impact on their overall QOL and social life [24]. Nguyen et al. argue that social events involving social eating no longer holds the same appeal [15]. The HNSCC survivor has to navigate between the risk of certain food textures being stuck in the throat, coughing, and aspiration in public, and therefore social eating can be intimidating and unappealing. Eating is also quite time consuming, as the food has to be processed in smaller bites and accompanied by plenty of liquid to aid the swallowing process, if the food texture is manageable at all [22].

The prospective study on QOL for patients with locally advanced HNSCC from Tribius et al. [13] found that social eating returned to baseline level within one year post-RT. These findings support the assumption that HNSCC survivors are able to adapt to a new life-style and accommodate to a new eating situation post-treatment, e.g., coughing after swallowing [22].

During swallowing, lubrication is a key component to ease the swallowing process. Xerostomia only contributes to aggravate the swallowing effort, regardless of age [25]. The study from Rogus-Pulina et al. [25] found that healthy individuals may refrain from eating or limit dietary intake concurrently with increased effort during swallowing. They suggested that application of a gel-based salvia substitute may ease the swallowing effort.

This study found that all study subjects suffered from xerostomia, based on the unstimulated sialometry test with a mean unstimulated flow rate of 0.1 ml/min. Therefore, adequate lubrication to aid the swallowing effort was needed. The test meals revealed that larger food items were more difficult to handle with xerostomia and required more lubrication compared to the smaller items. A few study subjects felt that they had inadequate lubrication to test the larger sized regular food items compared to the smaller sized, and refrained from testing the larger items.

Both dysphagia and xerostomia are life-changing conditions with no permanent solution yet to relieve the symptoms. In a previous study, a salivary stimulant (chewing gum) was found to stimulate residual saliva gland function and increase saliva flow for HNSCC [26]. Salivary substitutes come in many shapes such as sprays, gels, oils, mouthwashes, or viscous liquids, and are viscous products to be applied to the oral mucosa [27]. The substitutes primary consist of carboxymethyl-cellulose, polyethylene oxide, or animal mucins with the cellulose-based being most common in sprays and gels [28]. For this study, the gel-based salvia substitute was based on hydroxypropyl methylcellulose and polyethylene oxide. This composition was chosen to best resemble the lubricating and textural properties found in saliva, and to ease the swallow process and reduce muscularly fatigue while eating.

Limitations to this study include no validation or test–retest of the NRS scores for perceived swallowing difficulties. The self-reported assessment of dysphagia was based on the scoring of the EORTC QLQ-H&N35 questionnaire and not by objective measures like modified barium swallowing (MBS) or FEES. A more objective assessment of dysphagia could have added more information about the potential efficacy of the gel to the present study. Future studies ought to include objective measurement of the swallowing function MBS or FEES, and compare it to the self-reported measures of swallowing difficulties. This study did incorporate objective measurement of the saliva production with sialometry which should always be considered for evaluation of perceived dryness of the mouth. Future studies should also collect data on medication use and consider exclusion due to medication inducing xerostomia. It would be relevant to investigate whether a difference in dysphagia and xerostomia could be seen between older (< 70 years) and younger subjects treated with RT. This, however, was not the intention of this feasibility study and the variability in subjective measures of swallowing difficulties may be related to the small sample size and it could affect the investigated associations. Finally, the effect of the gel-based saliva substitute was immediate following application. A follow-up study should determine the length of the effectiveness of the gel and the interval for reapplication.

This feasibility study set out to challenge the texture of food items manageable for HNSCC survivors, knowing that the majority of the participants did not indulge in large regular food items on a regular basis. The food items selected for the test meals were chosen according to the variation in texture and level of difficulty [21]. Prior to testing, it was anticipated that the pureed item would not pose a problem, whereas the regular item most likely would be a challenge and a restriction for some participants. This assumption was proven correct (Fig. 3). The pureed substance was easy to process regardless of size or gel consumption, and the regular item was less manageable in the large version. The size of the food item did influence the oral intake. Consumption of some of the large-sized regular food items was avoided by some subjects, due to the risk of aspiration. Application of the gel did improve the oral intake of some of these items, but it never reached the threshold of the smaller sized items. The gel-based saliva substitute may be an aid towards a varied diet including regular food items, and can provide symptomatic relief for late dysphagia.

## Conclusion

This feasibility study showed reduction in self-reported dysphagia during a seven-day period for HNSCC survivors while eating six standardized food items. The immediate effect of the gel-based salivary substitute was measurable using the subjective NRS score, and showed a significant increase in mean NRS score for pureed and selected regular food items indicating less swallowing difficulties. Evaluation of EORTC QLQ-H&N35 showed improved QOL regarding less trouble swallowing and improved social eating after consumption of the oral gel.

## Electronic supplementary material

Below is the link to the electronic supplementary material.

Supplementary material 1 (DOCX 638 kb)
