# Supplementary material for: Reducing Late Dysphagia for Head and Neck Cancer Survivors with Oral Gel: A Feasibility Study
Source: Dysphagia. 2019 May 9;35(2):231–41. doi: 10.1007/s00455-019-10018-9 (PMC7136308; doi:10.1007/s00455-019-10018-9)
Supplement: Supplementary file 1 — Supplementary material 1 (DOCX 638 kb) [file 455_2019_10018_MOESM1_ESM.docx]

# Supplementary material


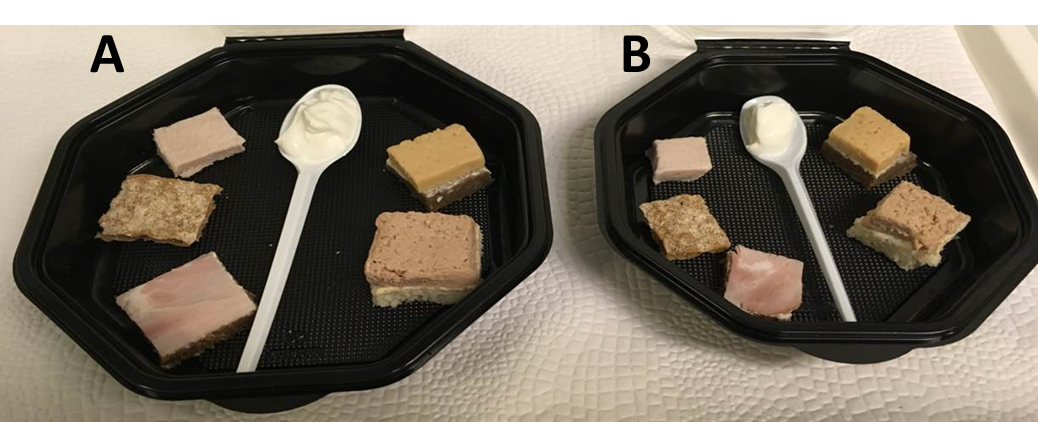


## Figure. 4 The test meal consisting of six large and small standardized food items

A Large food items: Yoghurt (spoon), Gratin with paté, White bread with paté, Rye bread with meat, crips bread and meat.

B Small food items: Yoghurt (spoon), Gratin with paté, White bread with paté, Rye bread with meat, crips bread and meat.
